# Difficulties in estimating the human burden of canine rabies

**DOI:** 10.1016/j.actatropica.2015.12.007

**Published:** 2017-01

**Authors:** Louise H. Taylor, Katie Hampson, Anna Fahrion, Bernadette Abela-Ridder, Louis H. Nel

**Affiliations:** a Global Alliance for Rabies Control, 529Humboldt Street, Suite 1, Manhattan, Kansas 66502, USA; b Boyd Orr Centre for Population and Ecosystem Health, Institute for Biodiversity, Animal Health & Comparative Medicine, University of Glasgow, Glasgow, United Kingdom; c Department of Control of Neglected Tropical Diseases, World Health Organization, Geneva, Switzerland; d Department of Microbiology and Plant Pathology, Faculty of Natural and Agricultural Sciences, University of Pretoria, Pretoria 0001, South Africa

**Keywords:** Canine rabies, Surveillance, Disease burden

## Abstract

Current passive surveillance data for canine rabies, particularly for the regions where the burden is highest, are inadequate for appropriate decision making on control efforts. Poor enforcement of existing legislation and poor implementation of international guidance reduce the effectiveness of surveillance systems, but another set of problems relates to the fact that canine rabies is an untreatable condition which affects very poor sectors of society. This results in an unknown, but potentially large proportion of rabies victims dying outside the health system, deaths that are unlikely to be recorded by surveillance systems based on health center records. This article critically evaluates the potential sources of information on the number of human deaths attributable to canine rabies, and how we might improve the estimates required to move towards the goal of global canine rabies elimination.

## Highlights

• Human rabies reporting is poor in endemic countries because of the disease’s neglect and fatality.
• Active surveillance shows that passive reporting systems can result in underreporting by up to two orders of magnitude.
• Our best estimates of the true burden of canine rabies come from modelling studies.
• Surveillance systems need to be strengthened and used in order for them to demonstrate their value.

## Graphical abstract

**Figure:**
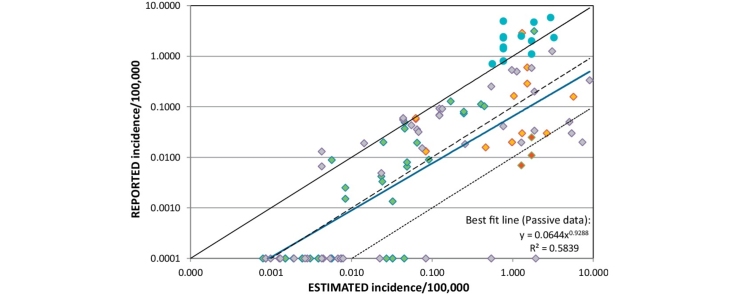

## 1 Introduction

When assessing the need for and priority of human health interventions, a basic requirement is to measure the impact of a given disease. The starting point is to find out how many people the disease affects and especially how many die from it. From this point we can begin to estimate how many years of productive life are lost to a disease, the consequent economic burden to a country, costs of preventative measures and how cost-effective an intervention might be. Unfortunately, when considering neglected tropical diseases, the assessment can fall at the very first hurdle. For canine rabies, we simply do not know how many people die in any given year for almost all countries where the disease is endemic.

Although, human rabies is officially a notifiable disease in the majority of rabies endemic countries, this is not sufficient to ensure effective surveillance data for many reasons (Taylor et al., 2015). Enforcement of legislation on the reporting of case data and information systems for collating case reports are frequently not available. Laboratory confirmation of suspected human rabies cases is very rarely carried out due to limited capacity and training, poor access to approved diagnostic tests and reagents and the difficulties of collecting the required post-mortem samples in rabies endemic countries (Banyard et al., 2013). Instead almost all human cases are diagnosed on clinical grounds, even though rabies may present in many ways (Suraweera et al., 2012) and misdiagnosis is common (Mallewa et al., 2007). Moreover, reporting of data from local to central levels is often incomplete due to limited use of reporting structures. Rabies case data reported to different authorities can conflict, for the same country and year of reporting, as found for Southern African countries reporting to the Southern and Eastern African Rabies Group, WHO and OIE databases (Nel, 2013). But perhaps the greatest challenges to accurate case reporting are that (i) canine rabies mostly affects the poorest sectors of society in the world's poorest countries and (ii) that rabies is a fatal disease. This combination means that most victims fail to access treatment, or return home to die having been advised that no effective treatment exists (Sudarshan et al., 2007, Taylor et al., 2015). These deaths, outside of health systems are not captured in surveillance systems based around health system records or in countries lacking civil registration systems to collect vital statistics on births and deaths (and their causes).

Given the absence of reliable surveillance data, where do we start in trying to assess the human cost of canine rabies in endemic countries? This paper seeks to critically assess the available surveillance and estimates of human deaths due to canine rabies and suggests ways in which these limited data can be improved upon to generate better information on which to base disease control decisions.

## 2 Using available passive surveillance data

The global collection of data on deaths from any neglected disease is a huge challenge, and early attempts to collate data for human deaths from canine rabies were no exception (Bogel and Motschwiller, 1986). Due to the lack of regular reporting of rabies cases to the World Health Organization (WHO) from many member states, the RABNET database was closed down in 2011 (WHO, 2013a), and has not yet been replaced. The World Organization for Animal Health’s (OIE’s) World Animal Health Information Database (WAHID) system is used to collect data on human cases of zoonotic diseases, as reported by veterinary health authorities (OIE, 2015), but the data is very incomplete (Table 1), and the frequent lack of intersectoral collaboration may mean that data reported by veterinary services do not accurately reflect health sector records.

Regional rabies databases are more successful. The Sistema de Información Epidemiológica (SIEPI) database across the Americas (Pan American Health Organization, 2015) is a well developed database, established in the 1970s, administered by PAHO (Belotto et al., 2005), and a critical part of canine rabies control across the continent. Reporting of data is relatively complete (in terms of the proportion of endemic countries reporting into the system) across Latin America and the Caribbean (Table 1) and has allowed detailed assessment of progress towards elimination (Vigilato et al., 2013). There are however, still some gaps from countries struggling with limited health infrastructure and capacity. A case in point is Haiti, thought to account for over 90% of the current human cases of rabies transmitted by dogs in Latin America (Hampson et al., 2015), but no human rabies cases were reported from there in 2013/4, and subsequent active surveillance there has further demonstrated how current systems under report rabies (Wallace et al., 2015). However, even for the countries reporting regularly, the level of detection of human deaths will depend on the surveillance capacity within the country.

The Rabies Bulletin Europe (RBE) database (Rabies Bulletin Europe, 2014) though voluntary, collects, collates and maps data on laboratory confirmed animal and human rabies cases from countries across Europe to assess progress of oral rabies vaccination efforts to eliminate wildlife rabies. Human rabies deaths in Europe are rare enough to attract significant media attention and extensive laboratory investigation to determine their precise origin, and therefore the RBE provides highly accurate records of human rabies deaths in Europe.

In contrast, across most of Africa and Asia, human deaths are much more common, rarely laboratory confirmed, and most often never captured by surveillance activities. Attempts to develop effective regional databases are beginning though, and country reports from regional rabies meetings have been compiled into datasets for Africa, the Middle East and Eastern Europe and Asia (Dodet and African Rabies Expert Bureau, 2009, Aikimbayev et al., 2014, Gongal and Wright, 2011, Searg, 2014, PARACON, 2015). Whilst these reports can provide insight into the issue, their current dependency on attendance at meetings makes these reports infrequent (annually at best) and data are usually not interpreted or compared to previous data (Dodet and African Rabies Expert Bureau, 2009, Aikimbayev et al., 2014).

There is variation amongst databases in frequency of reporting. Reports are submitted weekly to the SIEPI database, and quarterly to the Rabies Bulletin Europe. Whilst the main focus of these reports is monitoring of the rabies situation, timely information (at least within a month) is important to enable countries to enact control measures in the event of disease outbreaks.

Amongst canine rabies endemic countries a small number of governments regularly publish official data on human rabies deaths, for example India, Thailand, Sri Lanka and Nepal (Government of India, 2014b, Ministry of Public Health Thailand, 2013, Ministry of Health Sri Lanka, 2015, Department of Health Services Nepal, 2015). More often, reviews of multi-year surveillance data are published eg. in China (Yin et al., 2013, Song et al., 2014), Ethiopia (Deressa et al., 2010), India (Government of India, 2014a), South Africa (Weyer et al., 2011), and Turkey (Johnson et al., 2010). Data collation at the national level is important for the prioritization of outbreak responses and control program direction and analyses of national trends can reveal useful information about human cases over time, or areas where rabies risks are highest eg., (Song et al., 2014, Weyer et al., 2011), but such analyses are infrequent.

Besides the frequency of reporting, the quality of the reported data is a major concern. A recent survey identified a number of reasons for inadequate reporting, including (a) the challenges of reporting from remote areas, (b) inadequate follow-up of unconfirmed cases and confirmatory diagnosis, (c) inadequate financial investment in surveillance systems, (d) a lack of enforcement of existing legislation and guidance, (e) human rabies deaths occurring at home and outside the health system, (f) poor recognition of rabies by some health workers, (g) poor recognition of rabies' importance by politicians, (h) other competing health priorities, (i) lack of coordination between veterinary and medical authorities, (j) inadequate training of medical staff in rabies surveillance and case definitions and (k) a lack of understanding by bite victims on when and how to seek treatment (Taylor et al., 2015). At regional rabies meetings where surveillance data is shared, under reporting and the fact that deaths at home are often neither reported nor certified are widely recognized as significant problems (Dodet et al., 2015).

For these reasons, passive surveillance data for most canine rabies endemic countries is inadequate for accurately estimating the burden of disease, or the costs and benefits of control measures. Across canine rabies endemic countries we see a cycle of neglect—where there is no emphasis on control, there is no reliable data on how many people are affected, so there is no investment in control, and no progress is made (WHO, 2013b, Dodet et al., 2015). Passive surveillance for rabies generally only improves once control programmes are put in place, when awareness of the diseases is increased and good surveillance becomes necessary to assess effectiveness. A culture for reporting and sharing of data can develop and the value of these records can be widely seen, as in the Americas and Europe (Pan American Health Organization, 2015, Rabies Bulletin Europe, 2014). Hence, there are strong reasons to believe that surveillance databases will become increasingly valuable if canine rabies control efforts are initiated in currently endemic regions.

## 3 Utilizing local surveillance research

In the absence of reliable national statistics, research involving hospital-based and community-based surveys and epidemiological modeling has been carried out in a few countries (Kitala et al., 2000, Cleaveland et al., 2002, Hampson et al., 2008, Ly et al., 2009, Tenzin et al., 2011, Frey et al., 2013, Jemberu et al., 2013, Sudarshan et al., 2007, Suraweera et al., 2012, Sambo et al., 2013). Several studies have utilized an approach involving surveys of animal bite victims. Animal bites are an acute medical problem, likely to result in an interaction with health services, but there are still victims unable or unwilling to seek treatment overlooked by studies based at health facilities. Therefore, community-based studies involving interviews with bites victims and relatives of those who have died of rabies may produce more accurate estimates of rabies deaths. However memory recall attrition needs consideration for rabies (Sudarshan et al., 2007) as with other diseases (Allotey et al., 2015) as only the most recent incidents are likely to be accurately remembered.

In Machakos District, in Kenya, trained village residents carried out active surveillance by following up on all animal bites reported informally to them (not just through the health system). The study concluded that the annual incidence of human rabies deaths was 2.5/100,000 in the early 1990s (Kitala et al., 2000). Cleaveland et al. (2002) used active surveillance in the Mara Region in Tanzania, relying on monthly reporting of bites by key informants in communities and a series of probability calculations (a decision tree model) to estimate human rabies deaths. This study suggested the annual incidence of human rabies in Tanzania was 4.9 deaths/100,000 (Cleaveland et al., 2002).

Other studies have followed the decision tree approach of Cleaveland et al. (2002), though relying on health system records. Based on post-exposure prophylaxis (PEP) use and human death records from the Institute Pasteur Cambodia, which is the only source of free PEP in the country, an estimate of the incidence of suspected rabid dog bite injuries was derived (Ly et al., 2009). From a modified decision tree approach it was concluded that 810 human deaths from rabies occurred in Cambodia in 2007 representing an incidence of 5.8/100,000 (Ly et al., 2009). Based on decision tree modelling from hospital-based surveys, an annual human rabies incidence of 4.67 deaths/100,000 was estimated for the two rabies endemic areas of south Bhutan (Tenzin et al., 2011). In Chad, data collected from 50% of healthcare providers in the capital city, N'Djamena suggested an annual human incidence of 0.7/100,000 people (Frey et al., 2013). However, the studies based on passive collection of health records do not capture bite victims who do not enter the health system.

A more detailed field-based method used hospital records of people bitten by animals to initiate contact tracing in two districts of Tanzania. The 28 rabies deaths identified in these districts from 2002 to 2006 translated to average annual death rates from rabies of 1.5 and 2.3/100,000 population for the two districts respectively (Hampson et al., 2008). A subsequent study using the same methods from 2006 to 2009 revealed incidences of human rabies from 0.8–2.4 deaths per 100,000 population in an additional two districts (Sambo et al., 2013). In Ethiopia, an intensive longitudinal survey of households in the North Gondar zone during 2009–2010, recorded and followed up on any suspect human or domestic animal cases and revealed an incidence of 2.33/100,000 people (Jemberu et al., 2013).

In India, a multi-centric survey completed in 2003 employed an active search of records from 22 hospitals to identify recent ‘index cases’ of rabies deaths (the most recent human rabies deaths from rural and urban areas, recorded by the hospitals), and verified them by verbal autopsies (Sudarshan et al., 2007). These initiated community searches for other rabies deaths in the health center catchment areas and identified 235 rabies deaths. The annual number of clinically identifiable human rabies deaths across India was extrapolated to be 17,137, adjusted to 20,565 to account for atypical and paralytic forms of rabies, a rabies mortality rate of around 2/100,000 population. In a separate study, the analysis of more than 122,000 verbal autopsy reports from 2001 to 3 covering a representative sample of populations in India (part of the Million Death Study, an ongoing survey of deaths utilizing enhanced verbal autopsy techniques) detected 140 likely rabies deaths. Extrapolating across the whole of India, the authors concluded that in 2005 around 12,700 deaths resulted from symptomatically identifiable furious rabies, an incidence of 1.1/100,000 people, though this varied considerably across states (Suraweera et al., 2012). The methods used by Suraweera et al. (2012) like those used by Sudarshan et al. (2007) rely on diagnosis of rabies based on the characteristic symptoms of furious rabies, and are not expected to detect the paralytic form of rabies.

Such intensive research exercises can shed light on the extent of under reporting, for example between 1990 and 1996 in Tanzania, the mean number of officially reported human rabies deaths was 10.8 per year, corresponding to an annual incidence of 0.041 deaths/100,000 and suggesting underreporting by a factor of more than 100 (Cleaveland et al., 2002). Similarly, the Government of India officially recorded 274 deaths from rabies in 2005 and just 138 in 2013 (Government of India, 2006, Government of India, 2014a), well below the 728 cases recorded from just 22 infectious disease hospitals in 2002 (Sudarshan et al., 2007), and just 2% of the human deaths estimated for 2005 (Suraweera et al., 2012). Clearly even deaths recorded locally by hospitals are not reaching national databases. In contrast in Bhutan, the reported incidence of human rabies was 3.14/100,000, similar to decision-tree extrapolations from health facility records, indicating that under-reporting is much less of a concern here, perhaps due to the availability of free medical services and better access to hospitals (Tenzin et al., 2011).

Although local surveillance research is invaluable to assess the degree of under reporting in particular settings, it is impractical to carry out on a large scale. The study in Bhutan was carried out in an area known to be at higher rabies risk than the rest of the country (Tenzin et al., 2011), but often it is not well understood how representative study sites are. Results are therefore geographically and time limited, and although some extrapolation may be warranted, this must be done with caution. The variability in methodologies also significantly limits the degree to which direct comparisons can be drawn across studies.

Only one of these surveillance research studies directly addressed the proportion of bite victims who do not seek or do not obtain medical attention, which are critical determinants of human rabies mortality and its estimation. The use of contact tracing in Tanzania showed that between 15 and 24% of suspect rabies exposures did not seek medical attention, and of those that did, 14% did not obtain PEP (Hampson et al., 2008). These figures point to a sizeable under reporting factor (inherent where health system records are relied upon) that only intensive, community-based techniques can address.

## 4 Regional and global estimates derived from models

As the international health community moves towards a global elimination plan for canine rabies (FAO, 2013), it becomes necessary to assess the scale of the disease burden at a regional and global level to evaluate the benefits of global canine rabies elimination.

The first large-scale estimate of deaths from canine rabies was published in 2005, and focused on Africa and Asia (Knobel et al., 2005). This study used the probability decision tree approach developed in Cleaveland et al. (2002), starting with data on the human population of countries. It then applied the human: dog ratio and a number of probabilities (of being bitten by a dog, of that dog being rabid, of the person not receiving PEP, of an unprotected person developing rabies) to derive a number of people estimated to die from rabies in that country or region. Data for these parameters was sourced from published literature where available, and extrapolated to continental scales, ignoring variation between settings due to the lack of data. The figures reached were a global estimate of 55,000 human deaths per year (24,000 in Africa and 31,000 in Asia, Table 1). Shwiff et al. updated this study in 2013 (Shwiff et al., 2013), based on more recent population estimates and included Latin America, estimating just over 69,000 annual human deaths from canine rabies (Table 1).

A more detailed analysis of the global burden of canine rabies was recently undertaken by the Partners for Rabies Prevention (Hampson et al., 2015). This study, referred to hereafter as the PRP study used a similar probability-decision tree model, but estimated human deaths due to canine rabies in all countries of the world, using parameters derived from a wider variety of relevant sources, including recent published literature, available surveillance data, country expert surveys, and vaccine market data combined with international databases of population and development indices. Briefly, the model uses the product of bite incidence, the probabilities of (i) a biting animal being rabid, (ii) a bite victim receiving PEP, and (iii) in the absence of PEP, developing rabies, to extrapolate human rabies deaths. Functional relationships were estimated from empirical data to derive the first two parameters from information on dog vaccination coverage and relative reporting of deaths and PEP use, whilst other key parameters were based on published, but geographically limited datasets. The study concluded, based on 2010 data that canine rabies causes 58,991 (95% C.I. 25,000–159,000) human deaths a year (Table 1). This work identified changes from the results of the 2005 Knobel analysis, notably a dramatic increase in cases and use of PEP in China.

Mortality due to rabies was also calculated as part of the Global Burden of Disease (GBD) project, most recently carried out for 2010 (Lozano et al., 2012) and 2013 (GBD Mortality Causes of Death Collaborators, 2014). These studies, hereafter referred to as the GBD studies, relied on data mainly from vital registration (based on hospital records and including medical certification of the cause of death) and verbal autopsy (derived by standardized techniques from interviews with a close relative of the deceased) databases. This was supplemented where necessary with data from cancer registries, police and crime reports, burial and mortuary data, demographic and health surveys and censuses, and records of deaths in health facilities (Lozano et al., 2012). For rabies, a cause of death ensemble modelling (CODEm) approach was used. In this method, a large range of plausible statistical models are developed, compared and combined, with covariates retained or rejected based on ability to predict published data using out-of-sample predictive validity testing (Lozano et al., 2012 and references therein). Finally, deaths from individual cause of death models (for 235 causes) were adjusted to ensure that individual cause estimates summed to all-cause mortality for age-sex-country-year groups.

The GBD studies concluded that the number of human deaths caused by rabies (not differentiating canine rabies) was 26,400 (95% C.I. 15,200–45,200) in the 2010 study (Lozano et al., 2012), and 23,500 (95% C.I. 17,300–28,600) in the 2013 study (GBD Mortality Causes of Death Collaborators, 2014). Methodology adjustments between the two GBD studies revised the estimated human rabies mortality in 1990 from 54,100 (95% C.I. 32,400–103,400) calculated in the 2010 study to 38,400 (95% C.I. 26,700–48,700) calculated in the 2013 study, demonstrating the variation in outputs under different model assumptions. The narrower confidence intervals suggest more accurate estimates from the more recent analysis. Estimates for each individual cause of death from the GBD 2010 and GBD 2013 studies have been published by country, and rabies deaths in canine rabies endemic countries totaled 26,091 and 23,409 respectively (Table 1 for regional data, and references).

The WHO Department of Health Statistics and Information Systems also produced estimates for cause-specific mortality for each country for 2000–2012, based upon total mortality estimated from WHO life table death rates and resident populations estimated by the UN Population Division (WHO, 2014). Where possible these estimates relied upon high quality death registration data (vital registration records submitted to the WHO Mortality Database), disease-specific data sets (these were available for rabies only for China) and finally where no other reliable information existed for a country (applicable to most canine rabies endemic countries), cause fractions from the GBD 2010 models were applied to WHO and UN death rate estimates (WHO Department of Health Statistics and Information Systems, 2014). This method estimated a global total of 35,386 human deaths due to rabies in 2012 (WHO, 2014), of which an estimated 34,727 occurred in canine rabies endemic countries (Table 1).

## 5 Which information is the most useful?

Data from passive rabies surveillance is currently incomplete and in many endemic countries is of limited value in assessing the burden of canine rabies, or supporting control efforts alone. The most complete datasets are those where canine rabies has been eliminated or is close to elimination (Europe and the Americas), but the major canine rabies endemic areas of Africa and Asia have very poor reporting (Table 1, and Taylor et al., 2015).

In the few areas where active surveillance research has been carried out, this has demonstrated a high level of under reporting by passive surveillance systems. Active surveillance is likely to produce the best available estimates of human rabies deaths, but extrapolation beyond target areas may not be justified even within the same country, due to differences in population density, rabies endemicity and patterns of dog ownership (Suraweera et al., 2012, Tenzin et al., 2011). Standardization of active surveillance methodology would aid comparisons across countries and benefit regional control efforts.

With a few exceptions, model-derived estimates of human rabies deaths are the only measure of disease burden for canine rabies endemic countries. Mass extrapolation from limited data has its risks, but currently no alternatives exist for these countries. The most recent modelling studies which have produced global and country-specific estimates of human rabies deaths both rely on data pieced together from many sources and comparable data for every country is not available (GBD Mortality Causes of Death Collaborators, 2014, Hampson et al., 2015). Some parameters critical to the PRP model are supported by very limited available data, or modelled from indirect sources. The results are consequently very sensitive to errors in these parameters, for example in the proportion of bite victims able to access PEP, and the derived estimates of human deaths are very uncertain (Hampson et al., 2015).

Major regional varations in the quality of underlying data were also identified for the GBD study, and criticism of the necessary level of extrapolation from limited data is openly recognized (Lozano et al., 2012). Vital registration systems that include medical certification of the cause of death captured only about 18.8 million deaths of an estimated annual total of 51.7 million deaths globally in 2005 (Lozano et al., 2012). Currently, there are no vital registration statistics for sub-Saharan Africa, and the input data for these countries relies heavily on verbal autopsies, (appendix of GBD Mortality Causes of Death Collaborators, 2014).

Estimates of the burden of individual diseases have been criticized for their varying methods and because they are not constrained to sum to the total all-cause mortality, they may over represent specific causes of deaths (Lozano et al., 2012). However, there are reasons to suspect that both vital registration and verbal autopsy records underrepresent deaths from rabies. As many rabies victims die away from the health system, hospital records are less likely to include them and verbal autopsy records can only effectively differentiate rabies deaths if a history of an animal bite is included. Health and Demographic Surveillance Systems (Sankoh and Byass, 2014) which are standardized longitudinal surveys used in field sites around the world that provide estimates of death rates and cause of death through verbal autopsy techniques (Sankoh and Byass, 2014), do not typically include probing for dog bites. Without the history of an animal bite, rabies can be misclassified with other causes of neurological encephalitis, such as cerebral malaria (Mallewa et al., 2007). Many burden studies account for known underreporting with the use of expansion factors, but these need to be based on reliable evidence (Undurraga et al., 2013). As we have shown, there is a dearth of such studies for rabies, and the empirical and modelling work on the relationships between dog populations, rabies incidence and vaccination parameters and also on access to health care that is needed to estimate these factors is not yet sufficiently developed.

The major differences in methodologies are because the studies were designed to fulfill different needs. To assess health priority needs and compare across all causes of death for a country or a region, a standardized method such as the GBD study is necessary. However, the more detailed rabies specific data utilized in the PRP study would be expected to lead to more accurate predictions of the likely impacts of rabies control measures and therefore is more valuable for justifying country and regional based control programs.

## 6 Combining data sources

**Fig. 1:**
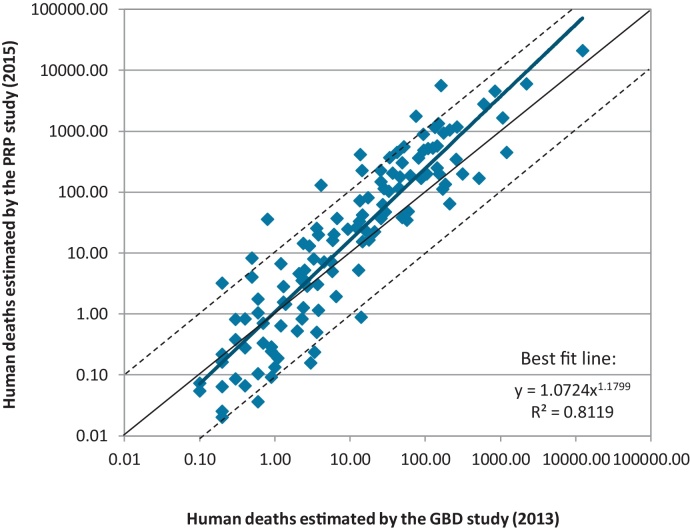
Comparison of national estimates of human deaths from the Partners for Rabies Prevention (PRP) study (2015) and from the Global Burden of Disease (GBD) study (2013), for the 122 canine rabies endemic countries only. The solid line represents 100% agreement, and the dashed lines represent estimates differing by a factor of 10. The bold line represents the best fit to the data.

Fig. 1 compares human death estimates for the canine rabies endemic countries from the two most recent comprehensive analyses, conducted by the PRP and the Global Burden of Disease study group (Hampson et al., 2015, GBD Mortality Causes of Death Collaborators, 2014). Given the varying methods and necessary extrapolation, there is reasonable correlation (*R*^2^ = 0.81) between the two sets of estimates. Compared to the GBD study (2013), the PRP study generally produces lower estimates for countries with little canine rabies infection and higher estimates for countries with a larger rabies burden. These trends could be explained by the inclusion in the GBD study of human rabies cases transmitted by wildlife species (a more significant factor in countries where canine rabies is a small problem), and by disproportionate under reporting of cases in poorer countries. In general however, these independent studies utilizing different methodologies suggest a broadly similar ranking of countries, and human rabies deaths almost all within an order of magnitude of each other across all countries.

**Fig. 2:**
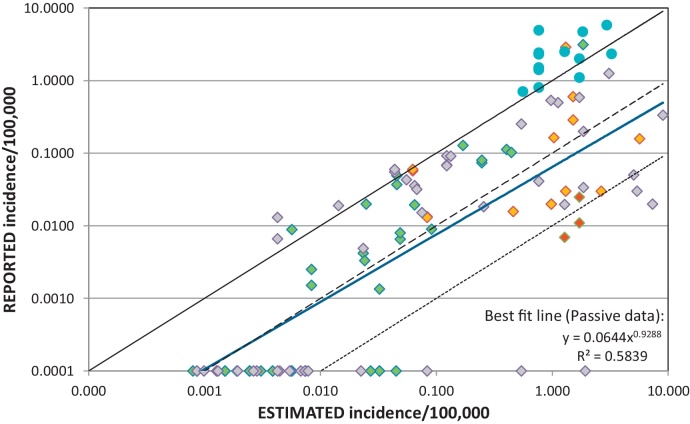
Comparison of reported incidences of human deaths in canine rabies endemic countries and estimated incidences from the PRP canine rabies burden study. Reported incidences(112 datapoints from 79 canine rabies endemic countries) are from the passive surveillance studies and online databases listed in Table 1 (diamonds), and the active and local surveillance research studies (circles) detailed in Section 3. Passive surveillance sources are colored according to the effectiveness of surveillance taken from a survey of human rabies surveillance systems (Taylor et al., 2015): Green =  surveillance system deemed effective, orange = surveillance system deemed ineffective, red = human rabies not a notifiable disease, grey = effectiveness unknown. The black lines represent the situation where 100% (solid), 10% (dashed) or 1% (dotted) of the estimated cases are reported. The bold blue line represents the best fit line through the passive datapoints only.

Comparing the country-specific estimates from the PRP study to surveillance data available provides some insight into the quality of the surveillance data (Fig. 2). Active surveillance studies produce incidences around or above the estimated incidences, which should be expected given that these studies were used as input parameters to the PRP study model. In contrast, passive surveillance systems (the vast majority of datapoints, which were not used directly in the model) result in human rabies incidences well below those predicted by the model, with reporting rates sometimes below 10% or even 1% (Fig. 2). The incidence data from countries with surveillance systems subjectively deemed as effective (Taylor et al., 2015) tend to fall closer to the estimated burdens, and those where human rabies is not a notifiable disease fall furthest away (Fig. 2).

## 7 Conclusions

Only regular, complete and reliable reporting of cases can provide a true picture of any disease situation, and high quality surveillance data on which to base disease management decisions (for canine rabies and other diseases) is needed. Current passive surveillance data is unreliable and incomplete for most canine rabies endemic countries and increased investment in surveillance should be prioritized by countries and international organizations to allow accurate evaluation of the need for and impacts of control programs.

Where active surveillance has been carried out, passive data collection for the same area demonstrates dramatic under reporting (Cleaveland et al., 2002, Suraweera et al., 2012). Active surveillance studies, even utilizing different methods, for the same country tend to produce more similar estimates of human rabies for countries with a high risk of canine rabies. For example, estimates of deaths from rabies in India were similar to one another at 2 and 1.1 deaths per 100,000, respectively (Sudarshan et al., 2007, Suraweera et al., 2012). Likewise studies from Tanzania derived relatively similar estimates from 0.8 up to 4.9 deaths per 100,000 from different parts of the country (Cleaveland et al., 2002, Hampson et al., 2008, Sambo et al., 2013).

Estimates at a global level come from two divergent studies and whilst these differ considerably (as expected given their limitations and different methodologies) they are of the same magnitude, countries rank roughly in the same order and the uncertainty (as shown by the confidence intervals around the estimates) in these estimates is overlapping. Whilst it may not be possible to be more precise, these comparisons give confidence that tens of thousands of people die from canine rabies each year across the world. This scale of disease burden, from an entirely preventable disease, should be enough to justify global investment in canine rabies control, of which reliable surveillance is a critical part.

In the short term, until the quality of passive surveillance systems across Africa and Asia improve, grounding of modelled estimates with active surveillance data, even if only available for a limited number of countries or time periods helps to build confidence in estimates. More active surveillance studies, ideally with standardized methodology are required to supply additional data with which to validate model estimates. However, in resource poor setting, innovative methods may be required and can be successfully implemented (Wallace et al., 2015) . One recent examination of a community-based passive surveillance system suggested that involving the entire population of Colombo City in Sri Lanka (assumed to be able to assess the whole dog population of the city) in reporting rabies cases could increase the sensitivity of passive surveillance to 100% even at a low (0.1%) disease prevalence (Craighead et al., 2015).

The 2005 International Health Regulations set standards for the surveillance of global health threats, and set a date of June 2012 for them to have been implemented by member states (WHO, 2008). Despite significant progress, many countries, particularly in Africa, Southeast Asia and the Eastern Mediterranean regions remain below the global average of 85% of targets achieved (WHO, 2015a). However, for polio, very high standards have been set for the completeness and sensitivity of surveillance (Global Polio Eradication Initiative, 2015), and most of the 29 countries with confirmed polio cases in recent years met these strict standards in 2013 and 2014 (WHO, 2015b). Increased priority for and better enforcement of surveillance across all diseases in Africa and Asia would benefit many stakeholders. They would allow better monitoring of health interventions and the earlier detection of emerging heath issues (Halliday et al., 2012).

International Health Guidelines (WHO, 2008) and new Terrestrial Animal Health surveillance guidelines (OIE, 2014) are available to help strengthen surveillance systems. The recently developed Rabies Surveillance Blueprint provides specific guidance on national rabies surveillance, based on international recommendations and practical experience, in a user friendly Question and Answer format (Partners for Rabies Prevention, 2014) and international cooperation through projects such as laboratory twinning and training exercises can significantly improve rabies surveillance (Zeynalova et al., 2014; Banyard et al., 2013, Wallace et al., 2015).

However, significant efforts need to be put into increasing the submission of samples from suspected rabies cases in animals and humans for laboratory confirmation. The perceived value of submitting information and diagnostic samples from animals is likely to play an important role here. If the submitter understands that human treatment decisions or control measures may result from their efforts, they may be encouraged to submit more information or samples (Halliday et al., 2012). On a larger scale, once rabies is targeted for control, surveillance will improve, because stakeholders have a reason to show the impacts of their efforts. This will typically mean that rabies incidence (based on passive surveillance data) will increase at the start of a control programme (as surveillance effort increases, Wallace et al., 2015) and then in the longer term will decline as control measures have an impact, which can then be quantified from the improved surveillance data. It is clear from Fig. 2 that effective control programs and effective surveillance systems go hand in hand (most countries with effective surveillance fall closer to the 100% reporting line, and have lower deaths from canine rabies than those without), and effective surveillance is critical to proving that elimination of human and animal cases of canine rabies has been reached.

Rather than being allowed to fall into disuse, regional and global reporting systems need to be strengthened and promoted, and improvements of the resulting data and their usefulness will follow. In this way, the control of rabies and other neglected diseases will receive the attention necessary to promote them from their status of neglect and surveillance to support control and elimination goals will be available.

## Figures and Tables

**Table 1:** Available surveillance data (A) and estimates (B) of annual human rabies deaths for canine rabies endemic countries. Figs. in square brackets are the number of countries for which information was available. Estimated deaths are given with 95% confidence intervals where available **Source of infection may include rabies from wildlife, ^$^countries reporting zero cases not captured. Classification of canine rabies endemic countries follows Hampson et al., 2015. (Total canine rabies endemic countries per region given in the header row). Discrepancies in country counts result from different country datasets across studies.

Year	Reference/source	Reporting/estimation Methods	Africa [48]	China [1]	India [1]	Rest of Asia [34]	All Asia [36]	All Asia + Africa [84]	Americas [16]	Africa, Asia + Americas [100]	Europe [22]	World [122]
(A) Surveillance data												
2013	OIE WAHID Zoonosis Database (OIE, 2015)	Surveillance reports from country reps** ^$^	1,268 [17]			336 [10]	336 [10]	1,604 [27]	13 [5]	1,618 [32]	18 [5]	1635 [37]
2013	SIEPI Database (Pan American Health Organization, 2015)	Surveillance reports from country reps							13 total, 6 from dogs [11]			
2013	Rabies Bulletin Europe Database (Rabies Bulletin Europe, 2014)	Surveillance reports from country reps**									10 [22]	
2013	SEARG Epidemiological database (SEARG, 2014)	Country reports at regional meetings	7 [5] South East Africa only									
2011	MEEREB 2013 meeting report (Aikimbayev et al., 2014)	Country reports at regional meetings				14 [2] Middle East only					9 [6] Eastern Europe only	
2008	AfroREB 2009 meeting report (Dodet and African Rabies Expert Bureau, 2009)	Country reports at regional meetings	146 [15] West Africa only									
2013	(Government of India, 2014a)	National surveillance data			138 [1]							
2005	(Government of India, 2006)	National surveillance data			274 [1]							
2012	(Song et al., 2014)	National surveillance data		1,420 [1]								
(B) Estimates of human cases												
2003	(Sudarshan et al., 2007)	Multi-centre community surveys and hospital records.			17, 137 (95%CIs 14,109–20,165) furious rabies. 20, 565 for all forms [1]							
2005	(Suraweera et al., 2012)	Verbal autopsies			12,700 (95% CIs 10,000 –15,500) furious rabies only [1]							
2003	(Knobel et al., 2005)	Probability decision-tree approach	23,705 (95% CIs6903–45,932)	2,336 (95% CIs 565–5,049)	19,713 (95% CIs4192–39,733)	9,489 (95% CIs2281–19,503)	31,539 (95% CIs8149–61,425)	55,270 (95% CIs 23,910–93,057)				
2012	(Shwiff et al., 2013)	Extension of Knobel 2005	31, 000				38,000		20	69,000		
2010	(Hampson et al., 2015)	Probability decision-tree approach	21,502 [48]	6,002 (95%CI1000–11,000) [1]	20,847 (95%CI7000–55,000) [1]	10,417 [34]	37,266 [36]	58,768 [84]	182 [16]	58,950 [100]	41 [22]	58, 991 [122]
2010	(Lozano et al., 2012, The Institute for Health Metrics and Evaluation, 2015) **	Global burden of disease ‘cause-of-death ensemble’ model	9,572 [48]	1,179 [1]	7,185 [1]	7,793 [34]	16,157 [36]	25,730 [84]	211 [16]	25,941 [100]	150 [22]	26,091 [122]
2012	WHO Global Health Estimates (WHO, 2014) **	Verbal autopsy, vital registration records and global burden of disease 2010 model outputs	16,816 [49]	2,635 [1]	7,437 [1]	7,291 [34]	17,363 [36]	34,179 [85]	337 [16]	34,516 [101]	211 [23]	34,727 [124]
2013	(GBD Mortality Causes of Death Collaborators, 2014) **	Global burden of disease ‘cause-of-death ensemble’ model	4,968 [49]	2,233 [1]	12,349 [1]	3,743 [34]	18,325 [36]	23,292 [85]	65 [16]	23,357 [101]	52 [22]	23,409 [123]
